# Investigation of Corticomuscular Functional Coupling during Hand Movements Using Vine Copula

**DOI:** 10.3390/brainsci12060754

**Published:** 2022-06-08

**Authors:** Fei Ye, JinSuo Ding, Kai Chen, Xugang Xi

**Affiliations:** 1 Department of Neurology, Affiliated Jinhua Hospital, Zhejiang University School of Medicine, Jinhua 321000, China; yf8800@163.com; 2 School of Automation, Hangzhou Dianzi University, Hangzhou 310018, China; dingjinsuo12345@163.com; 3 Hangzhou Mingzhou Naokang Rehabilitation Hospital, Hangzhou 311215, China; ckting928@gmail.com

**Keywords:** corticomuscular network, functional network, electroencephalography, surface electromyography, vine copula

## Abstract

Corticomuscular functional coupling reflects the neuronal communication between cortical oscillations and muscle activity. Although the motor cortex is significantly involved in complex motor tasks, there is still no detailed understanding of the cortical contribution during such tasks. In this paper, we first propose a vine copula model to describe corticomuscular functional coupling and we construct the brain muscle function network. First, we recorded surface electromyography (sEMG) and electroencephalography (EEG) signals corresponding to the hand open, hand close, wrist flexion, and wrist extension motions of 12 participants during the initial experiments. The pre-processed signals were translated into the marginal density functions of different channels through the generalized autoregressive conditional heteroscedasticity model. Subsequently, we calculated the Kendall rank correlation coefficient, and used the R-vine model to decompose the multi-dimensional marginal density function into two-dimensional copula coefficient to determine the structure of the R-vine. Finally, we used the normalized adjacency matrix to structure the corticomuscular network for each hand motion considered. Based on the adjacency matrix, we found that the Kendall rank correlation coefficient between EEG and EMG was low. Moreover, a significant difference was observed in the correlation between the C3 and EMG signals for the different hand-motion activities. We also observed two core nodes in the networks corresponding to the four activities when the vine copula model was applied. Moreover, there was a large difference in the connections of the network models corresponding to the different hand-motion activities. Therefore, we believe that our approach is sufficiently accurate in identifying and classifying motor tasks.

## 1. Introduction

Surface electromyography (sEMG) and electroencephalography (EEG) signals are known to most directly correlate with limb movement [1]. EEG signals contain information regarding the control exerted by the brain on the muscle [2], whereas the sEMG signal is the measurement of the potentials excited by motor neurons targeting the muscle fibers at the skin surface above the muscle, and thus, these signals directly reflect the motor intention of the human body [3]. Corticomuscular coherence (CMC) between EEG and sEMG is important for neuronal communication in peripheral and central sensorimotor systems [4]. The functional coupling between cortical oscillations and muscle activity is conducive to neuronal communication during motion control [5]. In this context, human–computer interaction research has extensively focused on the decoding of human motion intention via human bioelectrical signals, enabling machines to understand human motion intention, and developing a new generation of man–machine interfaces based on neural signals [6,7]. Moreover, the sensorimotor cortex usually produces oscillations related to muscle activity during simple muscle contractions [8]. Previous studies have also revealed that CMC reflects the communication between the motor neuron pool and cortex [9,10].

In particular, during hand gripping, the commands issued by the motor cortex are transmitted along the motor conduction path. Subsequently, the muscles and peripheral nerves of the upper limb are activated for motion to occur [11]. Proprioception synchronously occurs along the sensory pathway to the spinal cord, cerebellum, and part of the cerebral hemisphere. Most proprioceptive information is conducted to the sensory area of the brain for general analysis and the adjustment of movement commands [12]. The study of corticomuscular coupling can reveal the interaction between muscles and the cerebral cortex. This interaction demonstrates the information flow related to the cortex sending commands to muscles and the feedback of the muscle contractions. Therefore, a study of these interactions can contribute to understanding how the brain controls muscles, the influence of muscle activity on brain function, and the underlying nature of specific physiological conditions.

Limb motor dysfunction caused by stroke arises from damage to neuromuscular information-transmission pathways caused by brain lesions [13]. Previous studies have shown that the distal muscles of hemiplegic patients with stroke are significantly damaged, and that muscle functionality is harder to recover, that is, stroke patients are prone to slower hand movements and exhibit decreased movement accuracy and difficulty in grasping objects [14]. According to the relationship between the human cerebral cortex motor area, sensory area, and various parts of the body, the space occupied by the hand is the largest. Owing to the plasticity of the nervous system, the movement of the hand is conducive to activating the cerebral cortex, promoting blood flow and information transmission between the cerebral cortex and muscle tissue, and restoring the health of the neuromuscular pathways [15]. It has been reported in this context that the grasping movement of the hand can aid the rehabilitation of stroke patients [16].

The functional state of the human nervous system has become a new research hotspot in the field of motor dysfunction as an evaluation index of motor rehabilitation. The EEG signals of the brain motor cortex and EMG signals of the contralateral muscle tissue reflect the motor control information and the functional response information of the muscles to the brain’s control intention. Thus, the synchronous analysis of EEG and EMG signals can aid in estimating the functional relationship between the cerebral cortex and muscle tissue and describing the corticomuscular functional state [17].

Since Conway et al. [18] discovered CMC between cortical EEG and related EMG during movement in 1995, many researchers have begun to use different coherence-analysis methods to obtain the functional connection characteristics between the brain motor consciousness drive and muscle motor response. De Vries et al. [19] analyzed the coherence of the three muscles controlling the thumb and index finger for different pinch-force modes. They found that the coherence between muscles was enhanced at different frequencies during different motor tasks, and consequently, they concluded that the study of this coherence can reveal the neural origin of different muscle synergy patterns required for different tasks. Hu et al. [20] analyzed the intramuscular changes in related muscle pairs during wrist movements in healthy humans and found that the intermuscular coherence of a given pair of muscles was different in frequency and intensity for different movement modes. Fallani et al. [21] constructed a brain function network using the hand-motion-imaging EEG signals of patients with unilateral stroke. They found that the small-world characteristics and localities efficiency of unhealthy hand motion were significantly lower than the counterpart ones of healthy hand motion in a given frequency band during motor imagery tasks. Liu et al. [22] studied CMC between the upper arm muscle and motor cortex during isometric and isokinetic movements, and their results showed that the coherence of the band corresponding to isokinetic movement was far greater than that of isometric movement.

To better understand the functional interaction and information-transmission characteristics between the cerebral cortex and corresponding muscles, Granger causality (GC) analysis has been applied to the study of brain–neuromuscular synchronization [23,24]. A bidirectional (downlink EEG→EMG, uplink EMG→EEG) coupling was observed between EEG and EMG [12]. However, as the functional coupling between EEG and EMG involves nonlinear causality, the GC approach based on the established model cannot effectively describe the characteristics of nonlinear and higher-order coupling between EEG and EMG [24]. In related studies on motion recognition, the GC approach cannot effectively describe nonlinear characteristics and relying on linear characteristics alone may result in the inability to effectively distinguish human actions. Therefore, to overcome the afore-described problems, researchers have introduced a copula-based method for application to corticomuscular coupling analysis [25].

The copula theory was first proposed by Sklar [26] in 1959. The copula function has been used to establish a relationship between the marginal distribution probability function of each random variable and its overall joint distribution to predict the risk of high-dimensional markets. Dauwels et al. [27] established a Gaussian map model using copula and used multichannel EEG signals to infer the interaction between different brain regions.

Although the copula-based framework is universal, constructing a high-dimensional copula is difficult [28]. Therefore, Bedford [29] first proposed the vine copula model, which subsequently attracted wide academic attention. The vine copula model uses the vine structure to decompose the multidimensional copula function into corresponding sets of pair-copula functions. Vine copula can be used to construct flexible and diverse multivariate distributions, separate univariate from multivariate distributions in dependent structures, and solve the curse of dimensionality for parameter estimation under multivariate conditions. In this study, we used vine copula for nonlinear coupling analysis and the functional network modeling of EEG and EMG. The model decomposes multidimensional variables into multiple copula functions and their marginal distribution functions in the form of a vine; thus, the causality between brain and muscle multivariate variables can be obtained.

## 2. Materials and Methods

### 2.1. Vine Copula

Suppose that a N-dimensional random variable is $X=(X_{1},X_{2},\ldots ,X_{N})$, where its joint density function $f(x_{1},x_{2},\ldots ,x_{N})$ can be represented as

(1) $$\begin{array}{l} f(x_{1},x_{2},\ldots ,x_{N})=f_{N}(x_{N})•f(x_{N-1}|x_{N})\\ •f(x_{N-2}|x_{N-1},x_{n})\cdots f(x_{1}|x_{2},\ldots ,x_{N}) \end{array}$$

It can be seen from Equation (1) that it is necessary to obtain all the conditional densities; however, this is impractical for real-world application. In contrast, Sklar pointed out that a joint distribution can be decomposed into k marginal distributions and a copula function, which describes the correlation between variables [26].

Let F(•,⋯,•) denote a joint distribution function with marginal distribution functions $F_{1}(•),F_{2}(•),\ldots ,F_{N}(•)$. Consequently, there is a copula function F(•,⋯,•) that satisfies

(2) $$F(x_{1},x_{2},\cdots x_{N})=C(F_{1}(x_{1}),F_{2}(x_{2}),\cdots ,F_{N}(x_{N}))$$

The probability density function of distribution function $F(x_{1},x_{2},\cdots x_{N})$ can be expressed as:

(3) $$\begin{array}{c} f(x_{1},x_{2},\cdots x_{N})=c(F_{1}(x_{1}),F_{2}(x_{2}),\cdots ,F_{N}(x_{N}))\\ \prod_{n-1}^{N}f_{n}(x_{n}) \end{array}$$

where $c(u_{1},u_{2},\cdots u_{N})=\frac{\partial C(u_{1},u_{2},\cdots u_{N})}{\partial u_{1}\partial u_{2}\cdots \partial u_{1}}$, $f_{n}(•),n=1,2,\cdots ,N$ denotes the probability density function of distribution function $F(x_{1},x_{2},\cdots x_{N})$.

Vine copula can be used to solve the multidimensional correlation problem (i.e., there is a complex correlation between multiple random variables). The core idea of the vine copula function is to decompose the joint probability density function of the multidimensional random variable into a two-dimensional copula function about the original variable and its condition variable. The vine copula model [30] includes canonical vine (C-vine), drawable vine (D-vine), and regular vine (R-vine) categorizations. The nodes of each tree in the vine structure represent a distribution function or a conditional distribution function, and the edge represents the copula function used by the two nodes.

In the C-vine, only one node is connected to all the edges, while the other nodes are connected to one edge of the C-vine. In contrast, the tree of D-vine is linear. Unlike C-vine and D-vine, the structure of R-vine does not assume a fixed form. The structure is not predetermined but is structured according to the specific relationship of all the variables; therefore, different variables have different R-vine structures.

The density functions of R-vine can be expressed as:

(4) $$\begin{array}{l} f(x)=\prod_{i=1}^{n}f(x_{i})\prod_{j=1}^{n-1}\prod_{e\in E_{j}}C_{C_{e,a},C_{e,b}|D_{e}}\\ \;(F_{C_{e,a}|D_{e}}(x_{C_{e,a}}|x_{D_{e}}),F_{C_{e,b}|D_{e}}(x_{C_{e,b}}|x_{D_{e}})) \end{array}$$

where *j* represents the number of the tree, e={a,b} is the edge between nodes *a* and *b*, and $D_{e}$ is the set of variables contained in edge *e*; $C_{e,a},C_{e,b}|D_{e}$ represents the binary copula function between edges {*a*, *b*}.

The density function of each copula contains a pair of conditional distribution functions F(x|υ), which can be obtained by means of the following formula:

(5) $$F(x|\upsilon )=\frac{\partial C_{x,\upsilon_{i}}(F(x|\upsilon_{i}),F(\upsilon_{i}|\upsilon_{-i}))}{\partial F(\upsilon_{i}|\upsilon_{-i})}$$

where υ represents the *n*-dimensional parameter of the copula, $\upsilon_{i}$ is a component of υ, and $\upsilon_{-i}$ is the remaining *n* − 1 dimensional vector.

### 2.2. GARCH Model and Marginal Distribution

The autoregressive conditional heteroscedasticity (ARCH) model [31,32] can depict the time variation of conditional variance; however, the fitting effect must be sufficiently high to achieve the best depiction. For very high orders, parameter estimation becomes increasingly difficult. In contrast, the generalized ARCH (GARCH) model is the infinite-order ARCH model. Therefore, low-order GARCH(1,1) can be used to represent high-order ARCH model to reduce the difficulty of parameter estimation. For a time series $\left\{x_{1},x_{2},\cdots ,x_{n}\right\}$, the GARCH-t(1,1) model can be expressed as follows:

(6) $$\{\begin{array}{c} x_{t}=c_{0}+c_{1}x_{t-1}+a\\ a_{t}=\sigma_{t}\xi_{t}\\ \sigma_{t}=\omega +\alpha a_{t-1}^{2}+\beta \sigma_{t-1}+\gamma e_{t-1}^{2}L\\ \xi_{t}∼SkT(d,\lambda ) \end{array}$$

where $\omega ,\alpha ,\beta ,\gamma ,c_{0},c_{1}$, and d denote the model parameters, $a_{t}$ represents the volatility of the series, $\xi_{t}$ is the t-distribution with *d* degrees of freedom, and *L* is the indicator function that indicates the lag characteristics of variance changes under different conditions. The conditional marginal distribution expression of a single index can be derived from Equation (6):

(7) $$\begin{array}{l} F(x_{t+1})=P(a_{t+1}\leq x_{t}-c_{0}-c_{1}x_{t-1})\\ =P(\xi_{t+1}\leq \frac{x_{t}-c_{0}-c_{1}x_{t-1}}{\omega +\alpha a_{t-1}^{2}+\beta \sigma_{t-1}+\gamma e_{t-1}^{2}L})\\ =t_{d}(\frac{x_{t}-c_{0}-c_{1}x_{t-1}}{\omega +\alpha a_{t-1}^{2}+\beta \sigma_{t-1}+\gamma e_{t-1}^{2}L}) \end{array}$$

where $t_{d}(•)$ denotes the standard t-distribution function with *d* degrees of freedom. 

According to the maximum likelihood estimation, the parameter of the GARCH(1,1) model is estimated by using the sample $\left\{x_{1},x_{2},\cdots ,x_{n}\right\}$. Subsequently, the conditional marginal distribution function at time t + 1 can be obtained via Equation (7), and marginal density function $f(x_{t})$ can be obtained via the derivation of the distribution function.

### 2.3. Correlation Measure Based on Copula Function

With the established vine copula model, we can use the Kendall rank correlation coefficient to measure the correlation between the variables. The Kendall rank correlation coefficient measures the degree of agreement between two variables. Suppose that (X,Y) is a two-dimensional random vector, with $\left(X_{1},Y_{1}\right)$ and $\left(X_{2},Y_{2}\right)$ being independent and identically distributed. Consequently, the Kendall rank correlation coefficient τ of *X* and *Y* can be expressed as follows:

(8) $$\begin{array}{l} \tau (X,Y)=P((X_{1}-X_{2})(Y_{1}-Y_{2})>0)\\ \;\;\;-P((X_{1}-X_{2})(Y_{1}-Y_{2})<0)\\ =E(sign((X_{1}-X_{2})(Y_{1}-Y_{2}))) \end{array}$$

supposing that random variables *X* and *Y* satisfy the continuous marginal distribution and that their copula function is *C(u,v)*, we can write the rank correlation coefficient as follows:

(9) $$\rho_{\tau }(x_{1},x_{2})=4\int_{0}^{1}\int_{0}^{1}C(u,v)dC(u,v)-1$$

### 2.4. Modeling Step of Vine Copula

Based on the establishment of the GARCH model in Subsection B, we obtain the residual sequence of EEG and sEMG data. Subsequently, the residual sequence is transformed into a new sequence by the cumulative probability integral. In this section, we use the transformed sequence to model the vine copula. To select the optimal R-vine-copula function, the maximum spanning tree based on Prim (MST-PRIM) algorithm (see [33], chapter 23) is used to determine the R-vine structure as follows:

1. Calculate the Kendall correlation coefficients between all variables, compare the sum of absolute values, and choose the largest spanning tree as the structure of the first layer tree;
2. Select the optimal pair-copula function of the first layer tree structure by using the Akaike information criterion (AIC) and Bayesian information criterion (BIC), and calculate the conditional marginal distribution function;
3. According to step (2), calculate the Kendall correlation coefficients between all conditional variables, and set the generating tree with the maximum sum of the absolute values of all τ correlation coefficients as the structure of the second layer tree;
4. Select the second-level tree structure by using the AIC and BIC, and calculate the conditional marginal distribution function. Repeat steps (3) and (4) until only two nodes and one edge are left;
5. The decomposition expression of the joint density function of random variables is expressed by the marginal density function and pair-copula function.

### 2.5. Granger Causality

Correlative oscillations between the cerebral cortex and the muscles are not only conducted from the cerebral cortex where commands are issued to the muscles (descending), but are also fed back by the muscles to the cerebral cortex (ascending). Such oscillatory circuits allow the cerebral cortex to sense the functional state of the limb and integrate the flow of information from input to response. The Granger causality analysis method has been widely used in the field of neural network, which is defined as: adding the value of the x variable to the regression of y can improve the performance of the prediction, then it is said that x causes y, where the causal transitive relationship between x and y can be expressed as follows:

(10) $$G_{x\to y}=\ln\frac{S_{\mathrm{yy}}\left(f\right)}{H_{\mathrm{yy}}\left(f\right){\Gamma H}_{\mathrm{yy}}^{*}\left(f\right)}$$

(11) $$G_{y\to x}=\ln\frac{S_{\mathrm{xx}}\left(f\right)}{H_{\mathrm{xx}}\left(f\right){\Sigma H}_{\mathrm{xx}}^{*}\left(f\right)}$$

where $S_{\mathrm{xx}}$ and $S_{\mathrm{yy}}$ are the self-spectral density functions of x and y, respectively, Γ and Σ are the variances of $H_{\mathrm{yx}}$ and $H_{\mathrm{xy}}$, and the symbol * represents the transposed conjugate.

### 2.6. Computation of Network Characteristics

Path length shows the average number of the shortest path between every two vertices and is usually used to measure the global connectivity of networks. Characteristic path length is defined as follows [34]:

(12) $$L=\frac{1}{N\left(N-1\right)}\sum_{i\neq j\in \left[1,N\right]}l_{ij}$$

where $l_{\mathrm{ij}}$ is the shortest path length between vertices i and j.

In complex network theory, a clustering coefficient is a measure of the degree to which nodes in a network tend to cluster together. Clustering coefficient C is defined as follows [34]:

(13) $$C=\frac{1}{N}\sum_{i=1}^{N}C_{i}$$

(14) $$C_{i}=\frac{2E_{i}}{K_{i}\left(K_{i}-1\right)}$$

where N is the number of vertices, $C_{i}$ is the clustering coefficient of vertex $i,E_{i}$ is the number of vertex i and its neighbors that are actually connected with i, and $K_{i}$ is the degree of vertex i.

## 3. Experimental Procedure

In this study, six males and six females (age: 25 ± 2 years) were invited to participate in the experiments. We tested all participants using the Edinburgh Handedness Inventory (EHI) scale. The results showed that the mean EHI value for all participants was above 90 points, so the participants were right-handed. 

All the recruits had no history of major limb injury, and none of them had symptoms or signs of neuromuscular diseases. All participants provided signed informed consent according to the Helsinki Declaration, and all measurements were approved by the local ethics committee.

**Figure 1:**
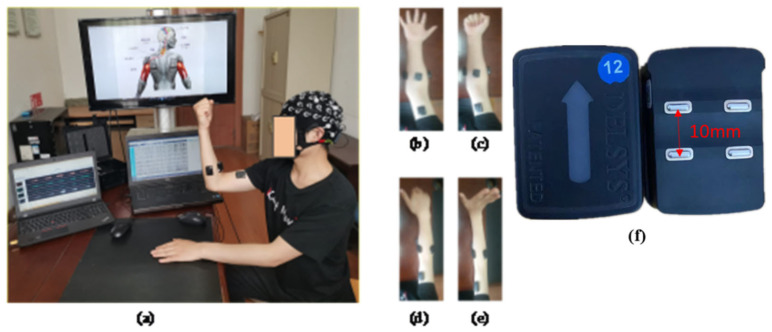
Setup for measuring motor tasks. (**a**) Experimental scene, (**b**) hand open, (**c**) hand closed, (**d**) wrist flexion, (**e**) wrist extension, (**f**) Delsys sEMG sensor diagram.

As shown in Figure 1a, the participants sat comfortably in a chair, kept their eyes closed, and performed specific hand motor tasks while focusing on imagining the motor task. Figure 1b–e show the four motor tasks were recorded: hand open (HO), hand close (HC), wrist flexion (WF), and wrist extension (WE). Hand open (HO) means placing your hand open and flat on a table, the whole hand is not forced, keeping it relaxed. Hand close (HC) means bending the fingers into a state of just making a fist on the basis of the Hand open (HO) state, that is, the hand does not exert force. Wrist flexion (WF) means pointing the fingers together and point to the ground, that is, the hand forms an angle of 90 degrees with the ground. Wrist extension (WE) means pointing the fingers together and point to the ceiling, that is, the hand forms an angle of 90 degrees with the ground.

**Figure 2:**
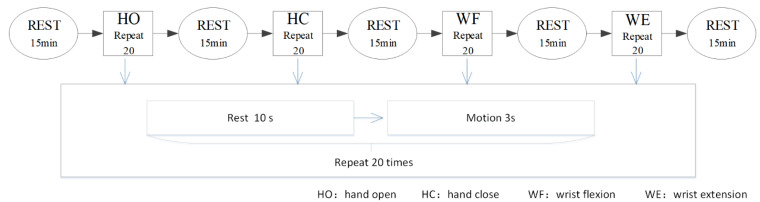
Experimental paradigms. HO: hand open, HC: hand close, WF: wrist flexion, WE: wrist extension.

The time of the experiment was fixed between 14:00 and 18:00 every day to avoid the influence of varying time factors. All participants were unaware of the experimental procedure prior to the experiment. During the experiment, the participants sat upright and relaxed their body while keeping the torso vertical. The upper limbs were naturally positioned to ensure that the electrode placements remained unchanged during the course of the experiment. In the process of signal collection, the participants only needed to execute the hand motion according to a prompt set in advance. The participants performed the tasks according to an operator’s instructions. First, we constructed a metronome for timing the experiment. As shown in Figure 2, before the start of each motion, the contestant kept resting and made a “ding” sound after about 10 s. At this time, the participants started and maintained the action until the second “ding” sound was made after 3 s. Then, the loop was entered for the next action, which was after a 10 s rest period. The participants could thus determine the beginning and end of the action according to the sound cues. Next, each participant was trained to complete the corresponding motion within the specified time. As shown in Figure 2, each motion was repeated 20 times and after every motion type was completed, the participant rested for 15 min to prevent fatigue. For one of the four actions of HO, HC, WF and WE, a complete motion took approximately 3 s, followed by relaxation for 10 s.

**Figure 3:**
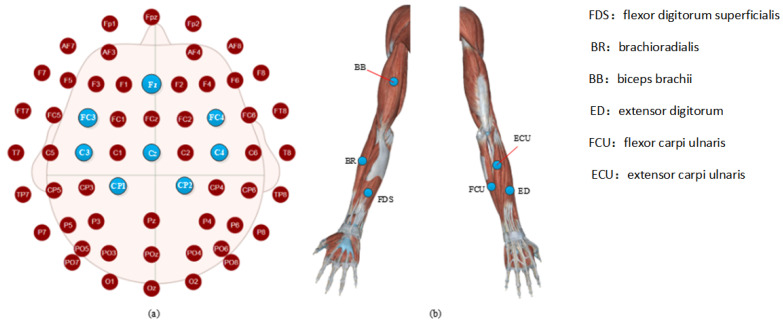
Electrode placement for (**a**) EEG, (**b**) EMG. FDS: flexor digitorum superficialis; BR: brachioradialis; BB: biceps brachii; ED: extensor digitorum; FCU: flexor carpi ulnaris; ECU: extensor carpi ulnaris. As shown in Figure 3a, EEG data were recorded by a digital EEG apparatus (g.MOBllab + MP—2015) at the following eight positions of the 10–20 systems: Cz, C3, C4, Cp1, Cp2, FC3, FC4, and Fz (Fpz was selected as the grounding electrode). Considering that all the subjects were right-handed, some of the electrodes were only placed in the left brain region to build a much leaner function network.

As shown in Figure 3b, the sEMG signals were recorded by using a TrignoTM Wireless EMG (Delsys Inc., Natick, MA, USA) device, which provides a 16-bit resolution, 20–450 Hz bandwidth, baseline noise of <1.25 uV, and n electrode distance of 10 mm. Six muscles’ sEMG signals were recorded using sensors 1–6 of the device, respectively. Sensor 1 recorded flexor digitorum superficialis (FDS), sensor 2 recorded brachioradialis (BR), sensor 3 recorded biceps brachii (BB), sensor 4 recorded extensor digitorum (ED), sensor 5 recorded flexor carpi ulnaris (FCU), and sensor 6 recording extensor carpi ulnaris (ECU).

These signals were sampled at 1000 Hz. Alcohol was used to wipe and clean the muscle area before electrode placement.

The EEG and EMG signals were collected synchronously by means of a trigger. The EEG signals were bandpass-filtered at 0.05–100 Hz, whereas the EMG signals were bandpass-filtered at 30–200 Hz. The EEG artifacts caused by blinking or eye movement were removed through independent component analysis (ICA). We rejected a total of two components during the analysis of the ICA algorithm. A 50 Hz notch filter was used to eliminate the power-line interference. The EMG signals were full-wave-rectified [35]. Finally, the signals were organized into a time series of 3000 data points (3 s).

## 4. Results

We separately analyzed the Kendall rank correlation coefficients of the EEG and EMG signals. Subsequently, we investigated the Kendall rank correlation coefficients across the different activities considered in the experiment. 

**Figure 4:**
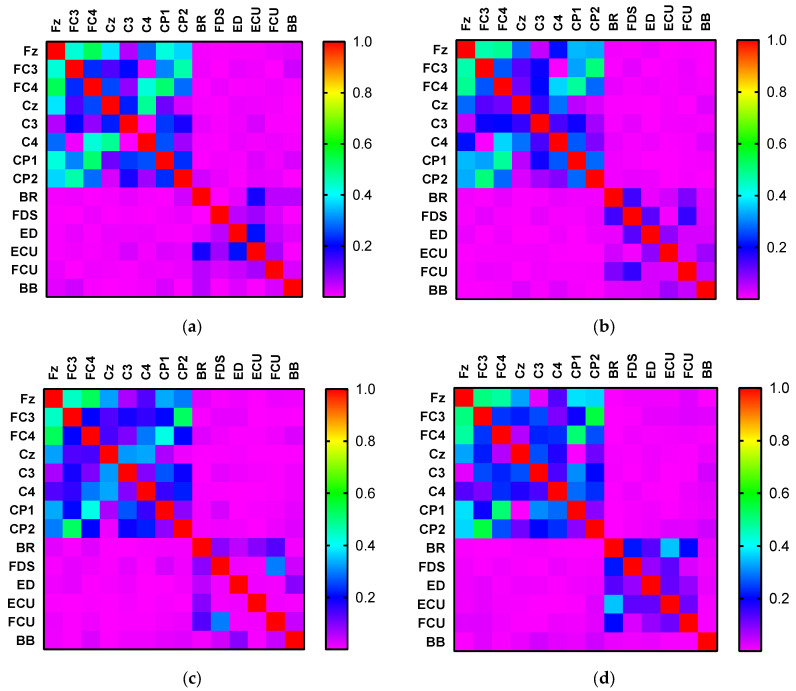
Heatmaps of the Kendall rank correlation coefficient of the EEG and EMG signals corresponding to (**a**) hand open, (**b**) hand closed, (**c**) wrist flexion, and (**d**) wrist extension.

Figure 4 shows the heatmap of the Kendall rank correlation coefficient of the EEG and EMG signals for the different activities, where the color according to the scale represents the coefficient size. 

**Figure 5:**
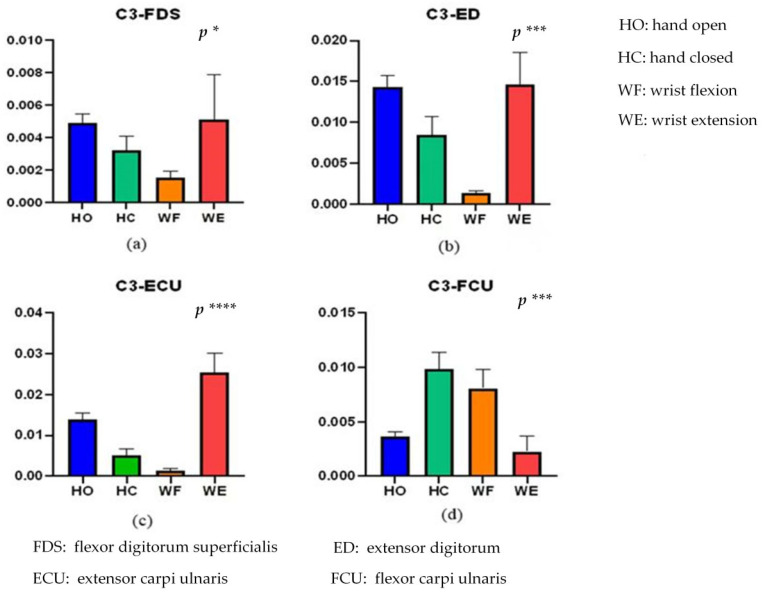
Kendall rank correlations of (**a**) C3-FDS, (**b**) C3-ED, (**c**) C3-ECU, and (**d**) C3-FCU for the four different activities. (* means *p* < 0.05, *** means *p* < 0.001, **** means *p* < 0.0001). HO: hand open, HC: hand closed, WF: wrist flexion and WE: wrist extension.

To evaluate the significant correlation difference for the different activities, we conducted a one-way analysis of variance (ANOVA). To evaluate the significant correlation difference for the different activities, we performed one-way ANOVA for each of the four conditions (C3-FDS, C3-ED, C3-ECU, and C3-FCU). After the normality test of the data, the different activities (HO, HC, WF, and WE) were independent variables, and the correlations of C3-FDS, C3-ED, C3-ECU, and C3-FCU for these activities were the dependent variables for a one-way ANOVA. The results for C3-FDS, C3-ED, C3-ECU, and C3-FCU are shown in Figure 5. 

From Figure 5, there is a significant correlation difference for the different activities (*p* < 0.05). Significant differences in activity of C3-FD, C3-ED, C3-ECU, and C3-FCU were (F(3956) = 3.012, *p* = 0.0293), (F(3956) = 5.57, *p* = 0.00086), (F(3956) = 7.284, *p* = 0.000078) and (F(3956) = 5.796, *p* = 0.00063), respectively. The C3-FDS correlation values of HO and WE are close to each other, and the value of WF is the lowest. In addition, C3-ED and C3-FDS exhibit similar results. The correlation difference of C3-ECU is more significant than that of the others (F(3956) = 7.284, *p* = 0.000078). Overall, we can distinguish these four activities through these correlations.

**Figure 6:**
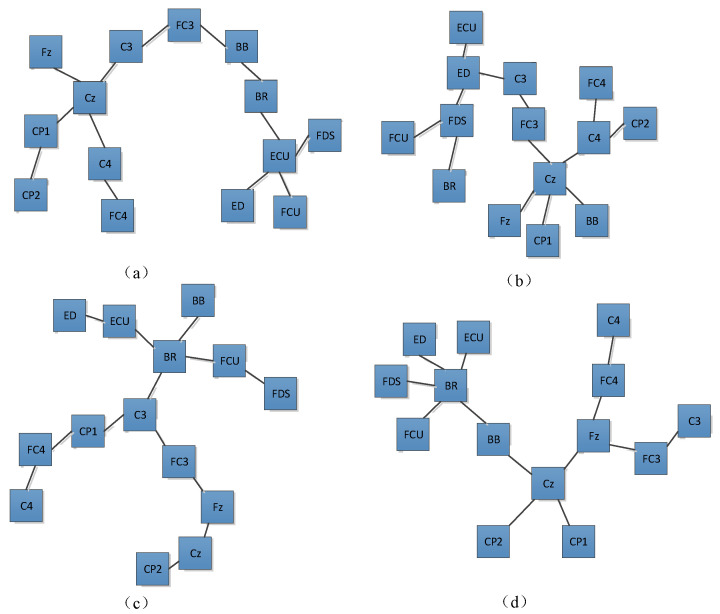
Vine copula first-layer tree structure corresponding to different activities: (**a**) hand open, (**b**) hand closed, (**c**) wrist flexion, (**d**) wrist extension. It can be observed from Figure 4 that the Kendall rank correlation coefficient between EEG and EMG is low for all the four activities. Nevertheless, the EEG signals are highly correlated. We also note that the correlation coefficient between the signals of the same type (EMG–EMG and EEG–EEG) is always higher than that between two different types of signals (EEG–EMG). Generally, the correlation between the Fz and other signals is strong, and the same is true for FC3 and FC4. For the HO and HC movements, a similarly strong correlation exists between the EEG signals. For WF and WE, the correlation between the EEG signals is similar. Meanwhile, the Kendall rank correlation of the FDS and FCU is higher than that of the other EMG channels for WF. However, in the case of WE, the correlation between BR and ECU is higher compared with that for other EMG channels.

To further understand the relationship between the channels from the brain and muscle, we constructed a vine copula structure for these motions. Figure 6 shows the results for the HO, HC, WF, and WE movements. From the figure, the EEG/EEG and EMG/EMG signals are linked together, with, in addition, channel BB being connected between EEG channels in the HC case. Channel FC3 is the communication channel between EMG and EEG for HO. Channel C3 is the communication channel between EMG and EEG for HC and WF. Channel Cz is the communication channel between EMG and EEG for WE. Core node means that this node is the node with the most connections to other nodes in the network. There are two core nodes for the different activities, for example, channels Cz and ECU are the core nodes for HO. For HO and HC, channels BR, FDS, ED, ECU, and FCU are connected. For WF and WE, channel BR forms the core node in the EMG. For HO, HC and WE, channel Cz forms the core node in the EEG.

**Figure 7:**
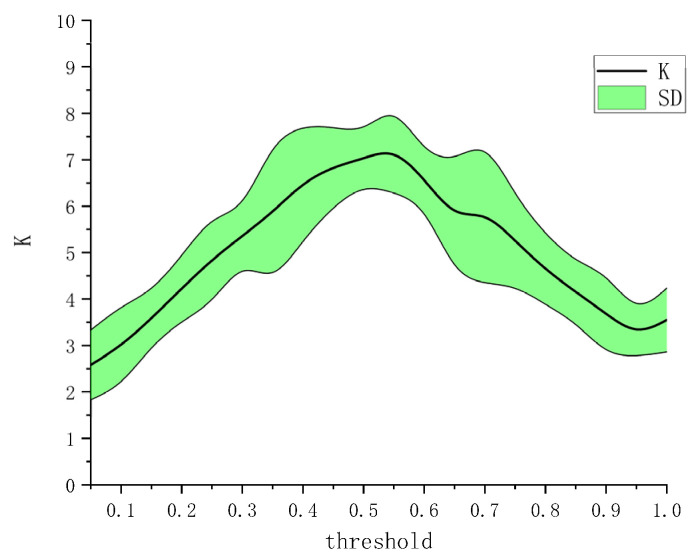
Determination of threshold. K: average node degree; SD: standard deviation of the average node degree.

From Figure 4, the EEG–EMG value is normally less than 0.05, whereas the EEG–EEG value is normally greater than 0.2 (even ~0.8). Moreover, the EMG–EMG value is normally less than 0.2. Owing to this difference, we normalized the EEG–EEG, EMG–EMG, and EEG–EMG values to [0,1], that is, dividing the EEG–EEG, EMG–EMG, and EEG–EMG values by EEG–EEG/EMG–EMG/EEG–EMG’s maximum values minus go to the minimum. The diagonal elements of the adjacency matrix were set to 0. The CMC could subsequently be mapped based on the normalized adjacency matrix. Here, it is crucial to determine the threshold for network connectivity, because different thresholds correspond to different network adjacency matrices. It is more flexible to set the threshold [36]. A previous study [37] showed that the optimal effect can be obtained when the average node degree K satisfies K≥2lnN, where *N* denotes the number of nodes. We chose a threshold of 0–1 step size of 0.05 for testing and observed the impact of different thresholds on building network adjacency matrices. We first calculated the average node degree [38] of all nodes in a single network of HO, HC, WF, and WE, and then averaged the calculated four node degree values to obtain the average node degree. We calculated the average node degree value of the network as a function of the threshold. The result is shown in Figure 7. Figure 7 shows that the average node degree is optimal when the threshold is at 0.55. Ultimately, the threshold was set to 0.55.

**Figure 8:**
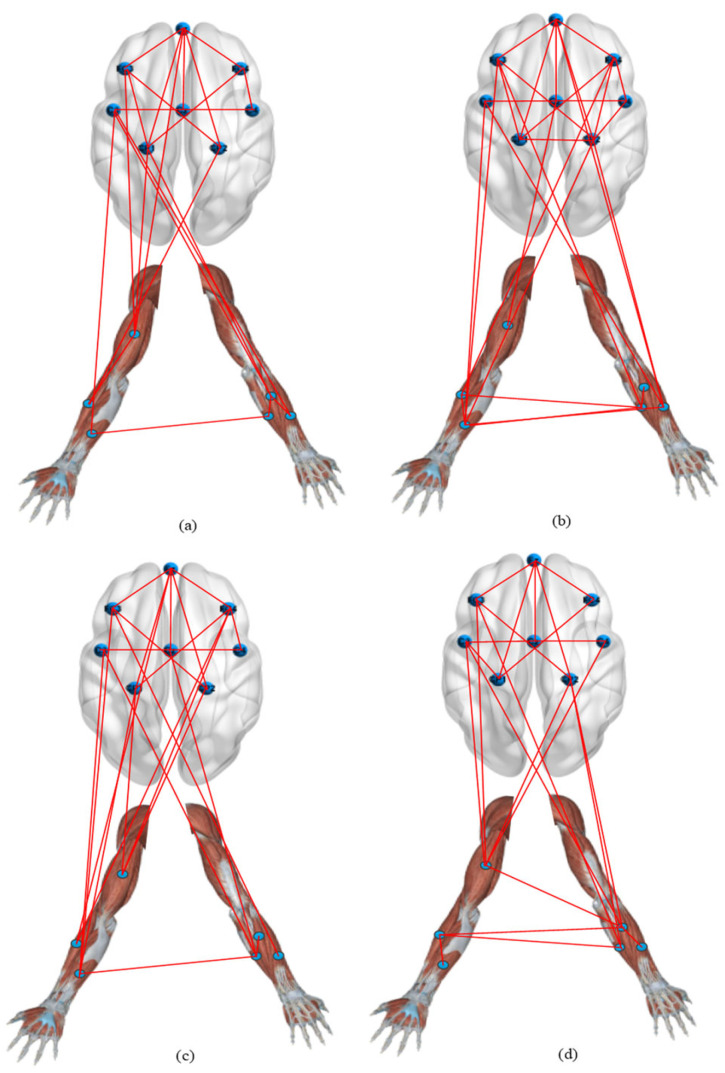
Corticomuscular network for different motor activities: (**a**) hand open, (**b**) hand closed, (**c**) wrist flexion, (**d**) wrist extension.

We obtained the corticomuscular function networks for the four different activities using this threshold. As shown in Figure 8, there are large differences in the connections of the network models corresponding to the activities.

**Figure 9:**
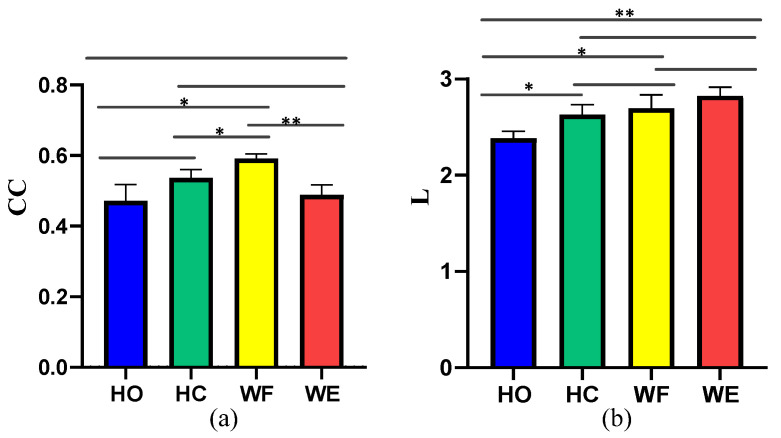

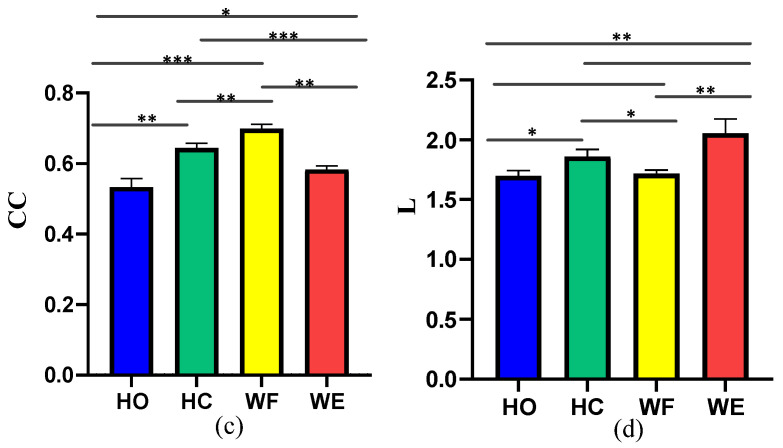
Clustering coefficient (CC) and characteristic path length (L) of the network for the different motor activities considered: (**a**) CC obtained with GC, (**b**) L obtained with GC, (**c**) CC obtained with vine copula, (**d**) L obtained with vine copula. (* means *p* < 0.05, ** means *p* < 0.01,*** means *p* < 0.001).

Moreover, we used the GC method to structure the corticomuscular function networks to evaluate the performance of the proposed method. To measure the statistical characteristics of the corticomuscular function network, we calculated the clustering coefficient (CC) and characteristic path length (L). A *t*-test was used to evaluate the significant difference between CC and L for the different motor activities; the results are shown in Figure 9. We observed a significant difference between the different activities when using the vine copula (*p* < 0.05). In contrast, with GC, the number of significant differences between the different activities is less. Compared to GC, the difference in the characteristics of the four activities obtained by using the vine copula model is more obvious. The CC value of the proposed model is larger than that of the GC model. The smaller characteristic path length indicates that the information-transfer efficiency of the proposed model is high. For the proposed vine copula model, it can be seen from the figure that the CC value of WF is the highest, which indicates that the network corresponding to WF is more complex than the other four motor task. The difference between HO and WF, HO and WE is larger than that for the other cases (*p* < 0.05). As regards the characteristic path length of the vine copula model, there is no significant difference between HO and WF and HC and WE (*p* > 0.05)

## 5. Discussion

In our research, we used vine copula to construct a brain–muscle coupling network model for the first time. The cortical coherence between EEG and EMG signals is important for neuronal communication in the peripheral and central sensorimotor systems. The brain–muscle coupling network we build helps us analyze the relationship between the cortex and muscles. Exploring the information interaction and functional coupling relationship between the brain and muscles under different conditions will help reveal the cooperative working mode of neural networks in the process of motion control from the system level. A visualized information transmission process between the cerebral cortex and muscle tissue must be established, and the state of cortical nerve function described.

Against this backdrop, the purpose of this study was to examine the corticomuscular coupling based on EMG and EEG recordings. In this context, the GC approach has been conventionally applied to the study of brain–neuromuscular synchronization. However, the approach suffers from certain limitations: first, it can only consider the causality of two time series and cannot be used for multivariate analysis. Second, it is also difficult to analyze the complex causality of nonlinear, non-Gaussian, and higher orders with the GC approach. Thus, Bezruchko et al. [39] proposed a nonlinear GC and used a p-order polynomial to construct an autoregression model. However, the prediction may be unstable when the dimensionality of the model or the order of the polynomial is increased for a short time series. Moreover, GC cannot distinguish whether causal relationships are direct or indirect. Spurious causality can appear between two processes when external sources are not considered [40]. To solve this problem, we proposed a vine copula model to describe corticomuscular functional coupling. The premise of underlying the use of this model is that the time series is transformed into a uniform distribution, and thus, the GARCH model is used to obtain the marginal density function of different channels. The MST-PRIM algorithm is used to determine the structure of R-vine copula. Figure 4 shows that the correlation coefficient between two signals of the same type (EMG–EMG and EEG–EEG) is always higher than that between two different signals (EEG–EMG). Moreover, from Figure 5, we find that there is a significant correlation difference for different hand motion activities, whereas Figure 7 and Figure 8 show that there is a significant difference in the brain–muscle network for different activities. These results illustrate that the model can also be used for the classification of motions. In Figure 6, two core nodes are observed for different activities as per the vine copula model. These results indicate that Cz may be the most tightly bound channel for the different activities considered. Furthermore, our results also show evidence of cortical activity related to human movement.

On this basis, this research can also be applied to the research of different types of brain diseases. Understanding the movement control process and the pathological mechanism of movement disorders also provides a new perspective for the evaluation of neurorehabilitation motor function status. By exploring the abnormal changes in cortical muscle coupling caused by diseases, it provides new judgment indicators for revealing the pathophysiological mechanism of brain diseases at the system level. On this basis, it also establishes neuromuscular coupling network imaging markers describing the disease, providing important auxiliary tools for the early diagnosis of patients and the evaluation of curative effects.

However, the study suffers from several key limitations that need to be addressed before the method can be applied for medical diagnosis or in rehabilitation medical robots under real-world conditions. We collected sEMG and EEG signals corresponding to specific hand motions from healthy young participants. Thus, we only studied the EMG–EEG functional coupling for healthy people. It is not known how well this model will work in a real scenario with unscripted free-form activities performed by patients or the elderly. Although there is no considerable difference between the characteristics of sEMG and EEG between participants with and without disabilities, the amplitude and frequency of the signal will still influence the correlation coefficient. These conditions need to be investigated before applying the proposed model for clinical purposes.

In future, we plan to continue studying corticomuscular functional coupling and explore in detail the relationships between cortical oscillations and muscle activity. Moreover, we plan to apply the method to the recognition of daily actions and the rehabilitation of stroke patients.

## 6. Conclusions

In the process of motor control, the electroencephalogram of the cerebral motor cortex and the electromyogram of the contralateral muscle tissue reflect the motor control information and the functional response information of the muscles to the brain control intention. In this study, we propose a rattan connection model to describe the cortical-muscle functional coupling. We constructed brain muscle functional networks. Then, the preprocessed signals were transformed into marginal density functions of different channels by a generalized autoregressive conditional heteroskedasticity model. Subsequently, we calculated the Kendall rank correlation coefficient and used the R-vine model to decompose the multidimensional marginal density function into two-dimensional Copula coefficients to determine the structure of the R-vine. Finally, we use the normalized adjacency matrix to construct the corticomuscular network for each hand movement considered. The results showed a low Kendall rank correlation coefficient between EEG and EMG. Furthermore, significant differences were observed in the correlation between C3 and EMG signals for different hand motor tasks. When applying the vine copula model, we also observed two core nodes in the network corresponding to the four activities. In addition, the connections of the network models corresponding to different hand actions are quite different. Therefore, we believe that our method is sufficiently accurate in recognizing and classifying motion tasks. This study helps to reveal the cooperative working mode of neural network in the process of motor control.

## Data Availability

The data generated and/or analyzed during the current study are not publicly available for legal/ethical reasons but are available from the corresponding author on reasonable request.
